# Women’s experiences of midwifery care immediately before and after caesarean section deliveries at a public Hospital in the Western Region of Ghana

**DOI:** 10.1186/s12884-019-2698-4

**Published:** 2020-01-02

**Authors:** Agani Afaya, Veronica Millicent Dzomeku, Elizabeth A. Baku, Richard Adongo Afaya, Mavis Ofori, Samuel Agyeibi, Frederick Boateng, Rosemond Ohwui Gamor, Elsie Gyasi-Kwofie, Prudence P. Mwini Nyaledzigbor

**Affiliations:** 1 School of Nursing and Midwifery, University of Health and Allied Sciences, Ho, Ghana; 2 Department of Nursing, Kwame Nkrumah University of Science and Technology, Kumasi, Ghana

**Keywords:** Caesarean section, Midwife, Midwifery care, Mothers delivery experience

## Abstract

**Background:**

Childbirth remains a uniquely multifaceted, mental-cognitive and a major life experience to women. It is composed of a variety of psycho social and emotional aspects and creates memories, sometimes bad experiences and unmet expectations which leaves the mother with lasting scars. Therefore, this study aimed at exploring post-caesarean section delivered mothers experiences of midwifery care in a public hospital in Ghana.

**Methods:**

This descriptive exploratory qualitative research used an interpretative approach to explore mothers’ experiences of midwifery care immediately before and after caesarean section (CS). The study employed a purposive sampling technique in recruiting 22 participants who had knowledge of the phenomenon under study. Data collection was guided by an interview guide, which involved face to face individual interviews and focus group discussion at the postnatal ward and clinic. All interviews were audio-recorded and lasted 30–40 min. Audio recordings were transcribed verbatim and inductive thematic data analysis employed.

**Results:**

Four major themes emerged from the analysis of participants’ transcripts: Support by Midwives (physical and psychological, and attitude towards patients’ pain management); Protection of mothers (provision of privacy, confidentiality and physical environment); Provision of information/communication (before caesarean section, and before a minor task) and midwives’ attitude (attitude towards delivery care).

**Conclusion:**

Mothers delivered by caesarean section had varied experiences of midwifery care which were both positive and negative ones. Provision of psychological support and adequate pain management were positive experiences. The challenges experienced were related to provision of information, privacy, and physical support. Participants, who underwent emergency CS in particular, were dissatisfied with the provision of information concerning the surgical procedure. Provision of privacy and physical support were also issues of great concern. We therefore, recommend supportive and sensitive midwifery care particularly for mothers undergoing emergency CS. Documenting women’s diverse experiences of midwifery care before and after CS delivery is important to healthcare providers, hospital managers and policy makers as the feedback garnered can be used to improve maternity services and inform decisions on midwifery care.

## Background

Improvement in the quality of health care delivery is an essential priority worldwide, and the aim of improving health care quality is to guarantee patient safety, and improve clinical outcomes thereby reducing disease burden [1, 2]. Provision of quality of care and ensuring patients’ satisfaction has been a challenge faced by many healthcare facilities across the globe [1]. Exploring the quality of midwifery care from the patients’ perspective is an important part of quality of health care assessment [1].

Ghana as a country has made several steps in improving health care delivery especially maternal and child health care. In 2003, delivery care fees were abolished in the northern part of Ghana (northern, upper east and west regions) and the central region. This was further extended to the remaining six regions in 2005. In July 2008, the free maternal health care policy was implemented under the National Health Insurance Scheme (NHIS). The free maternal health care policy covers antenatal care, delivery services, postnatal care and 3 months’ neonatal care under the mothers’ NHIS card [3]. Since its implementation, maternal health care utilisation (antenatal care and supervised deliveries) has shot up [4, 5]. Midwives practice in both private and public sectors within the country. Ghana now has about five teaching hospitals, 267 hospitals, 137 district hospitals, 1003 clinics, 855 health centres, and 4185 CHPS. These health facilities provide maternity services to mothers and patients [6].

Childbirth remains a uniquely multifaceted, mentally-cognitive and a major life experience to women [7, 8]. It is composed of a variety of psychosocial and emotional aspects and creates memories, sometimes bad experiences and unmet expectations which may leave the mother with memories for life [8]. These experiences may influence their desire and attitude towards future pregnancy and delivery. Childbirth comes with excitement, however, for some women, this excitement is dampened as a result of physical pain, especially for those women who have had a caesarean delivery [9]. In recent times, there has been a great upsurge in the rate of caesarean delivery among women in developed and developing countries [9, 10]. A World Health Organisation (WHO) report revealed that, between 1990 and 2014 the global average of caesarean section rate increased from 12.4–18.6% [11]. Other studies done in developed countries revealed varied CS rates (12–86%) [12–15] while developing countries reporting 2 to 39% [12, 16–20]. This increase in CS rates across the globe has reflected in Ghana. From 1990 to 2005, Ghana documented an increased CS rate between 4.5 and 6.4% [21]. According to the Ghana Demographic Health Survey 2014, CS deliveries increased to 13% [21]. Despite the upsurge of CS rates and medical advances, most women still hold the view of the importance of actively participating through the labour process to achieve a vaginal birth [9, 10]. Women in developing countries often prepare psychologically and physically for spontaneous vaginal delivery. In the event that the unexpected happens, the women are left with lower self-esteem and a feeling of hopelessness [22]. Some women perceive that a previous CS may lead to subsequent caesarean sections. This leaves the woman with a sense of disappointment and loss of control leading to distrust in her personal abilities as a childbearing woman [22]. Studies also indicate that CS deliveries lead to high rates of post-traumatic stress symptoms [23, 24]. The incidence of this disorder is higher among women who have gone through emergency caesarean section or an instrumental delivery compared to elective caesarean section or normal vaginal delivery [24].

Midwives are responsible for providing care and support to women during pregnancy, labour, and delivery. The quality and manner of providing midwifery care during the delivery process contribute to a positive or negative childbirth experience [25]. The role and responsibilities of midwives at this critical stage of a woman’s life may lead to diverse outcomes ranging from life to death and from health to physical injuries, with significant effects on the mental, and emotional health of the mother and child [26]. In low and middle-income countries, maternity care may be compromised by mistreatment during childbirth, including abusive, neglectful, or disrespectful care. Several studies have indicated that women may refuse to seek maternity care when they have previously been disrespected and may discourage other women from seeking maternity care even if the provider (midwife) is skilled in managing complications before and after delivery [27–30].

Some studies have been done on women’s experiences with maternity services in Ghana [25], however, little is known about women’s experiences of midwifery care immediately before and after CS delivery. Therefore, the current study aimed to explore the experiences of post-caesarean delivered mothers at a public hospital in the Ellembelle district of the Western region of Ghana. Such data is important to healthcare providers, hospital managers and policy-makers as the feedback garnered can be used to improve maternity services and inform decisions about midwifery care.

## Methods

### Research team and reflexivity

The research team comprised of seven nurses who had interest in improving maternal and child health care. VMD, EAB and PPM are professionals with a background in midwifery/obstetrics. All interviews were conducted by either AA, MO, SA, and supervised by VMD, EAB, PPM who had previous interviewing experience during their Masters and PhD studies. The interviewers were registered nurses with clinical experience in maternal and child health care but were not workers in the hospital under study. Prior to the interviews, none of the subjects were known to the interviewers, either personal or professional capacity.

### Research design

The study employed a descriptive exploratory qualitative design and used the interpretative approach to explore mothers’ experiences of midwifery care immediately before and after caesarean delivery. This design was appropriate as it afforded the nurse researchers the opportunity to focus on gaining a deeper understanding of an experience and to investigate the meaning of experiences related to issues that have implications for nursing practice and research [31]. The study was conducted using individual interviews and focus group discussion. The individual interviews produced more detailed, sensitive information and offered more insight into participants’ personal thoughts, feelings and world view [32]. The focus group was employed due to its interpersonal and interactive nature which allows participants to produce information that might not be gathered from a single participant [32, 33**]**.

### Study setting

The study was conducted at St. Martin De Pores Hospital – Eikwe. Eikwe is a small coastal town located along the Sanzule – Atuabo main road in the Ellembele district. The hospital was opened in the mid-1930s as a Maternity Home and Orphanage. It has since grown to become a General hospital with a bed complement of 175 and provides services ranging from clinical, preventive, curative, rehabilitative, surgery and health support. Obstetrics and Gynaecology is the mainstay of the hospital. The Maternity Ward has the highest bed capacity of 63. St Martin De Porres Hospital serves as a referral centre for the district.

### Sampling technique and sample size

A purposive sampling technique was employed in recruiting participants who had knowledge of the phenomenon under study. Data saturation was reached at the 18th interview and confirmed by the focus group when no new information was obtainable even with probing [34]. There were no refusals or drop outs from the study.

### Data collection procedure

Data were collected through face to face interviews with mothers lasting between 30 and 40 min. A semi-structured interview guide that was available in English and Twi was used to explore mothers’ experiences of midwifery care. Two midwives at the maternity ward and PNC assisted in recruiting participants (recruitment links). The authors had prior discussions with the ‘link midwives’ to spell out the purpose of the study as well as the inclusion and exclusion criteria before the interviews. The study protocol and participant information leaflets were handed over to them to serve as reminders. The participants were approached after discharge by the ‘link midwives’ to introduce the study to them. When a potential participant was identified, the first author was informed through a phone call. The first author then screened potential participants for eligibility and maintained confidentiality. Eighteen individual interviews and one focus group discussion were conducted from August to September 2018. The main research question asked was “*Please can you share with me your expectations and experiences of midwifery care before and after your CS”?* Prompt and probing questions were used for further explanation following participants’ responses. The interview sessions had a moderator and an assistant moderator, especially for the focus group discussion.

### Data analysis

Data were analysed using inductive thematic analysis and was done concurrently with data collection [35]. Transcripts were read severally to deduce meaning out of participants’ views. The data were managed manually. Preliminary themes were established and followed in subsequent interviews and substantiated with field notes to completely develop themes. The first author analysed the data and confirmed by the second and third author to ensure the participant’s views were accurately and sufficiently represented; with variations discussed. The discussions were done to resolve differences and build consensus on identified themes that required further exploration in subsequent interviews. To determine the exactness of the individual interviews, the key participants’ responses were summarised and its validity confirmed with participants after each interview.

### Trustworthiness

Approaches adopted to ensure trustworthiness included the use of the same interview guide which was piloted before its use. An audit trail was kept for other scholars to validate the processes undertaken in the study. A detailed description of the research setting, methodology (COREQ criteria were used) [36] and background of the sample have been provided to allow for transferability of the findings in similar contexts [37]. In-depth interviews allowed full exploration of the midwives’ characteristics that influence CS delivered mothers’ experiences of midwifery care. Concurrent data analysis ensured that the women’s comments were further explored in subsequent interviews. Data was later crosschecked with participants after the interviews to verify their responses.

### Ethical consideration

Ethical approval to conduct the study was obtained from the University of Health and Allied Sciences Research Ethical Committee (UHAS-REC A.10 [34] 17–18). Participants signed a consent form after agreeing to participate and were informed that they could withdraw from the study anytime. Participants were assured of anonymity, privacy, and confidentiality of their responses. The interviewees were assigned codes (001–022) to ensure anonymity. Audio files were transferred from the recorder to the principal investigators’ laptop and locked with a password.

## Results

Twenty-two mothers were interviewed. The average age of participants was 28 years. All the participants were married, and the majority were aged between 21 and 35 years and primiparous. Half of the participants attained either primary or senior high school education. Also, the majority of participants had emergency CS delivery (see Table 1).

**Table 1:** Sociodemographic Characteristics of Participants

Variables	Number (N)
Age	
≥ 20	2
21–35	16
36–50	4
Religion	
Christianity	20
Islam	2
Educational status	
No formal education	2
Primary/SHS	10
College	7
University	3
Occupation	
Housewife	2
Farmer	3
Business	7
Civil servant	2
Fishmonger	8
Type of family	
Nuclear	16
Extended	6
Parity	
Primipara	13
Multipara	9
Type of CS	
Emergency	16
Planned	6

Nine subthemes emerged from data analysis which was classified under four major themes. These major themes were; Support by Midwife; Protection of mothers; Provision of information/communication; Midwives attitude (see Table 2).

**Table 2:** Core Themes generated during Data Analysis

	Major Themes	Sub-themes
1.	Support by Midwife	∙ Physical support
		∙ Psychological/emotional support ∙ Attitude towards patients pain management
2.	Protection of mothers	∙ Provision of privacy
		∙ Maintain confidentiality ∙ Physical environment
3.	Provision of information/communication	∙ Before CS ∙ Before a minor task (procedure)
4.	Midwives attitude	∙ Attitudes towards delivery care

### Support by midwife

#### Physical and psychological support given by midwives

Some participants appreciated midwives’ psychological support (reassurance) before caesarean section, the majority reported that they had misconceptions (such as; you might die or will not have normal vaginal delivery again) about CS but the midwives demystified their misconceptions. A few mothers felt that they were not psychologically prepared for the procedure.

Most women were reassured by the midwife

“*The midwives were helpful, they gave me emotional support before I was sent to the theatre and assured me of safe operation…and after returning from the theatre they were all around me, I felt at home in the hands of midwives”* (Participant 4; Planned CS)

*“I heard that you could die from caesarean section. My fears were allayed after explanation of the procedure to me by the midwife”* (Participant 5; Emergency CS)

Though most of the women were reassured by the midwife, there was an exception where a woman felt abandoned;

*“I was told by my friends who delivered through caesarean section that it was painful, and that made me anxious, but my anxiety was never allayed by any midwife. I was left to my faith”* (Participant 1; Emergency CS)

Some participants had high expectations from midwives, however, they were inadequately supported during physical activity (massages, warm baths, breathing techniques, mobilisation and holding hands).

*“Midwives did not provide the support (warm bath and mobilisation) which I most expected because I knew they understand and have much information about the procedure so I expected them to support me physically but that never happened. But thanks to my ‘mum’ whom I had support from…”* (Participant 18; Emergency CS)

Most mothers narrated that they did not receive physical support from midwives, as pain brings about limitations of some activities after every surgical procedure.

“…*it was difficult and excruciating, so one woman just decided to assist me out of the bed but the midwife shouted at her to stop and let me do it by myself…”* (FG Participant 2; Emergency CS)

Though most mothers said they were not physically supported during activities, there were few exceptions where mothers thought the midwives were overwhelmed by the number of women in the ward.

*“I think they are overwhelmed with the work because they are always few (2) on duty compared to the number of women in the ward…especially in the night… they can’t attend to everyone at the same time”* (Participant 11; Planned CS)

#### Pain management by midwives (midwives attitude towards pain management)

Most of the mothers felt midwives were able to manage their pain effectively after surgery. They expressed that midwives were prompt to their call and were present to assure them of pain relief.

A participant narrated

*"…I felt intense abdominal pain after I recovered from the sedation during the surgery…immediately I complained it was managed with painkillers by the Midwife. …she did everything possible to relieve me of the pain”* (Participant 12; Emergency CS)

Though most mothers were satisfied with how their pain was managed, few expressed dissatisfactions with pain management. Some mothers complained that midwives intentionally allowed them to go through the pain and only used verbal information to relieve pain, which was not possible.

According to a participant, pain management was inadequate, she narrated

“…e*ven though I was in pain after surgery, I wasn’t given any pain medication. They just told me that as for operation it is painful so I should just try and cope…”* (FG Participant 18; Planned CS)

### Provision of protection by midwives

#### Provision of privacy

Most mothers, particularly those who had emergency CS were embarrassed as the proper protection of their privacy was not maintained. The majority were exposed to other patients and relatives during physical examinations especially vaginal examination. Though the ward did not have enough screens, midwives could have limited the way they were exposed.

*“Because it was an emergency, the midwife did the shaving on my private part in the room whiles I was on the stretcher without any screen… It was my mother who pulled the door curtain so that someone walking outside will not see me…” (FG, Participant 2; Emergency CS)*

Although some participants were not satisfied with the provision of privacy, others were satisfied.

*“They covered me with a screen when they were examining me before the operation and when they were passing my intravenous line”* (Participant 18; Planned CS)

#### Confidentiality

Almost all participants had confidence in midwives keeping secrets. Participants reported that they could confide in midwives with their information. A woman narrated;

*“I am very confident that midwives will not share my information with anyone, ever since I was admitted I have not heard the midwives telling people about my history or any information that I gave them…I think they are very secretive about mothers’ information…”* (FG, Participant 3; Planned CS)

#### Physical environment

The majority of mothers expressed their views about the poor nature of the hospital/ward environment. Some mothers were worried about the odour emanating from a placenta pit outside the ward. Others complained about the untidy nature of the ward environment and the consequent infestation with flies. Non-availability of mosquito nets and subsequent exposure to mosquito bites was another issue of grave concern.

A mother narrated

“…*there are too many flies within the ward, the cleaners do not clean the place well...there are also many mosquitoes here, notwithstanding the stinking smell from the ‘placenta pit’ behind the building is very offensive…”* (Participant 4; Planned CS)

Few mothers said the hospital/ward environment was clean and friendly.

A participant narrated

“…*the environment is good and friendly to me, and I like the hospital/ward environment…it really looks like a hospital”* (Participant 6; Emergency CS)

#### Information provision/communication by midwives

Provision of information about procedures carried out on a client is essential for the understanding and cooperation of patients. According to some participants, midwives explained every procedure that was performed during the preparation for surgery.

*“When they were to examine and shave my private parts, everything was explained before it was commenced and I was not exposed to other patients…in fact I like the way midwives communicated to me, it was very polite”* (Participant 11; Planned CS)

Some participants, those who underwent emergency CS in particular, were dissatisfied with the provision of information concerning the surgical procedure. The lack of sufficient information provided by midwives increased their fear of the outcome of the surgery. The majority anticipated assurance from midwives that the outcome of the procedure will be good to allay their anxiety however, this was not met.

A participant said

*“The midwives did not explain anything about caesarean section delivery and its complications to me or my family. All they said to my mother and me was that they have to perform the operation on me to deliver my baby. How the operation was going to be performed, what I will go through, where they will do the operation, nothing was communicated to me. The only information I had before the surgery and was holding on to was what my friends and other people told me…that the surgery is perilous and painful …”* (Participant 2; Emergency CS)

#### Midwives attitude towards patients’ care

Mothers had bad opinion of midwives prior to attending the facility for delivery services, perceiving midwives as being rude and heartless. Contrary to this perception, the majority of mothers reported having good and positive experiences with midwives. A mother shared her experience;

*“…I heard that when you fail to push or comply with their directives, they will shout at you or beat you up but that was not the case with me…they were young midwives and very respectful…”* (Participant 17; Emergency CS)

Although the majority of the participants expressed that the attitude of midwives was good, a participant felt midwives were impatient and harsh.

*“There was this particular midwife who was very harsh and had bad communication skills. …when I reported to the maternity unit, I met this midwife and told her I was having pains in my loins so the doctor asked me to come here… I didn’t even finish talking, and she started shouting that if you have pains is this the place to come for medication…she didn’t allow me to explain myself… so I further said my cervix is tied, so the doctor asked me to report here…she started yelling…so why didn’t you tell me that when you came in…”* (FG participant 2; Emergency CS)

## Discussion

This study explored mothers’ experiences of midwifery care immediately before and after caesarean delivery. Various birth experiences were used by participants to express their birth stories of midwifery care. The provision of physical and emotional support during delivery has a significant influence on maternal satisfaction with the entire birth experience. When this aspect of care is neglected particularly during caesarean section delivery, it could lead to increased anxiety of the expectant mothers. However, given the necessary attention during this period, could impact the outcome of the surgery positively. Most participants’ in this study expressed their satisfaction with the psychological (emotional) support given by midwives. The present study finding is counter-intuitive to a study conducted in the Limpopo Province in South Africa, where it was observed that midwives offered limited emotional support during childbirth [38]. The South African study reported inadequate number of midwives during a shift which may have accounted for the variation. Our finding is consistent with a study done in Jordan where it was observed that some women did not receive sufficient physical activity support from midwives but had better support from female relatives during delivery [39]. It is observed that limited midwives in developing countries [40] might have contributed to the similarities between these studies, where a midwife would have to attend to more than one woman at a time during labour. Mothers who are not adequately assisted by midwives in the course of delivery relatively may report a negative birth experience [39]. It is therefore recommended that midwives provide the needed physical support during the delivery process in order to prevent negative birth experience. There is also an urgent need to increase the number of health personnel (midwives) to meet the physical and emotional needs of mothers as some indicated inadequate midwives per shift.

Provision of privacy is a key requirement of women utilising maternal care services, especially for physical examinations as well as the delivery process itself [41]. A sense of shame is usually attached to the process of physical examination and procedures like perineal shaving [41]. In the current study, the majority of mothers were humiliated during the performance of procedures resulting from inadequate seclusion. Women in prior studies have reiterated the need to provide privacy during physical examinations and procedures [42–44] by shielding them from other women or visitors [45]. Though the ward had few screens, midwives could have done better in the provision of privacy during physical examination of the private parts (perineal area) of mothers. It is recommended that midwives pay more attention around the provision of privacy when dealing with emergency cases.

A clean ward environment is an essential consideration for the future use of a facility [46]. Mothers in the current study expressed that the hospital/ward environment did not meet their expectations as some made mention that the environment was not good for their health and that of the newborn. The study recommends an improvement in the ward environment by removing the placenta pit near the ward and providing sufficient mosquito nets for mothers and their babies.

Some women in the present study provided several examples of situations in which the midwives communicated with them effectively. They highlighted the importance of midwives showing interest in their wellbeing, and providing clear explanations to procedures. This is in line with prior studies where women reported being satisfied with the information they received from midwives [7, 47] and recommended that effective communication and relational skills of healthcare personnel are major determinants of trust between care providers and women [7, 47]. Midwives need to be more proactive in the provision of information and explanations to procedures to ensure effective and cooperative maternity care, as some participants, particularly those who underwent emergency CS were distraught with the inadequate provision of information about the surgical procedure.

It was observed in this study that midwives were respectful and kind during the delivery process. This is inconsistent with findings from a qualitative study in Tanzania [48] where women reported being disrespected and abused by midwives in the course of their delivery. Promoting respectful maternity care is well recognized across the globe as one of the key measures aimed at enhancing utilisation and quality maternal care [49]. Women, therefore, seek respect while receiving maternity care [41] as participants in this study alluded to. Health care provider behaviour and attitudes are, therefore, major determinants of utilisation of skilled maternity care [41]. We therefore, recommend that midwives intensify efforts at promoting respectful maternity care.

### Strengths and limitations

Comparatively, the studied participants were highly educated and this is a limitation at the same time a strength. Educated participants may be more empowered to speak out about their experiences (either negative or positive) than their less-educated counterparts. This suggests that the women in our study may have shared a more precise picture of the degree of their experiences than a representative sample would have. However, the less educated mothers were far less than forthcoming compared with those reported in previous studies. This infers that we could have omitted some important data about the opinions of less educated mothers from rural backgrounds. We acknowledge this as a vital limitation since none of the educated mothers were ready to speak out for the less educated participants.

The majority of participants were emergency CS deliveries and with their critical conditions, they might have had few contact hours with the midwife before CS which may influence their experiences of midwifery care. With emergency situations, the midwives will exhibit a high sense of professionalism in order to save the woman’s life and after a successful CS delivery, the mother might not be able to critique the same midwife that expedited her successful CS delivery. Moreover, we acknowledge that there may have been some bias given that most of the women in our sample had emergency CS. It is possible that the expectations and views of emergency CS delivered mothers may be very different from those who had elective CS.

Nonetheless, the current qualitative health research used the grounded theory where the full iterative cycle was performed: after few interviews preliminary themes were established, on the basis of which the researchers returned to the field and new interviews were conducted until data saturation was achieved. Triangulation between the results of literature studies and the data analysis of the interviews has heightened the validity of this research.

## Conclusion

From the analysis, four major themes were found: (1) Support by Midwife, (2) Protection of mothers, (3) Provision of information/communication and (4) Midwives attitude. Mothers shared diverse experiences of midwifery care including both positive and negative ones. Provision of psychological support, midwives’ attitude, confidentiality and adequate pain management were positive experiences. A number of challenges were experienced by mothers which were related to the provision of information, privacy, and physical support. Participants, those who underwent emergency CS in particular, were dissatisfied with the provision of information concerning the surgical procedure. This has implications for the provision of quality maternity care. Particular sensitivity around provision of privacy and information by midwives may be required during performance of procedures before CS. These areas may require special attention by midwives to ensure high-quality care and subsequent utilisation of services provided by the facility. We recommend supportive and sensitive midwifery care particularly for mothers undergoing emergency CS.

Future research should focus on exploring experiences of emergency CS deliveries from the perspectives of midwives.

## Data Availability

The datasets used and/or analyzed during the current study are available from the corresponding author on reasonable request.

## Abbreviations

CHPS: Community-based health planning and services
CS: Caesarean section
FG: Focus group
NHIS: National Health Insurance Scheme
PNC: Postnatal clinic
WHO: World Health Organisation
